# Earthquake Disaster Risk Perception Process Model for Rural Households: A Pilot Study from Southwestern China

**DOI:** 10.3390/ijerph16224512

**Published:** 2019-11-15

**Authors:** Dingde Xu, Yi Liu, Xin Deng, Chen Qing, Linmei Zhuang, Zhuolin Yong, Kai Huang

**Affiliations:** 1 Sichuan Center for Rural Development Research, College of Management of Sichuan Agricultural University, Chengdu 611130, China; 2 College of Management of Sichuan Agricultural University, Chengdu 611130, China; lyx1@stu.sicau.edu.cn (Y.L.); qingchen@stu.sicau.edu.cn (C.Q.); zhuanglinmei@stu.sicau.edu.cn (L.Z.); zhuolinyong@stu.sicau.edu.cn (Z.Y.); hk666@stu.sicau.edu.cn (K.H.); 3 College of Economics of Sichuan Agricultural University, Chengdu 611130, China; dengxin@sicau.edu.cn

**Keywords:** risk perception, risk reduction, risk awareness, disaster avoidance behavior, Wenchuan earthquake, China

## Abstract

There are many important factors to consider when creating robust, regional disaster prevention systems. These include rural households’ knowledge and reported skills of earthquake disasters, disaster risk perception, awareness of disaster risk reduction, willingness to purchase insurance, and willingness to relocate to avoid disasters. However, few empirical studies have systematically established the theoretical research frameworks to analyze these factors. This study analyzed the data sampled from 241 rural households located in counties affected by the 2008 Wenchuan earthquake. A theoretical model was designed to investigate rural households’ disaster risk perception and used path analysis to systematically analyze the mechanism of the factors stated above. The results showed that 53.11% of rural households had a stronger willingness to purchase disease insurance and 72.19% had a stronger willingness to relocate to avoid disasters. Risk perception, knowledge and reported skills, and awareness of disaster risk reduction were significantly correlated with a willingness to purchase disaster insurance. Risk perception and awareness of disaster risk reduction were significantly positively correlated with a willingness to relocate to avoid disasters. Knowledge and reported skills indirectly affected the willingness to purchase insurance and the willingness to relocate to avoid disasters through risk perception and awareness of disaster risk reduction. Risk perception could indirectly affect the willingness to purchase insurance and the willingness to relocate to avoid disasters through awareness of disaster risk reduction.

## 1. Introduction

Earthquakes, landslides, mudslides, and other disasters have seriously threatened the lives and property of Chinese residents [1,2,3,4,5]. Since 2000, there have been 179 earthquakes in China greater than magnitude 5.0, causing a reported 488,400 casualties and direct economic losses of 1.13 trillion Yuan [6]. People living in poverty are often the most vulnerable to the devastating effects of earthquakes [7,8].

It is important to prevent and manage the effects of disasters in advance to help reduce the loss of life and property damage [9,10,11,12]. Many empirical studies have shown that the implementation of disaster insurance and relocation are effective ways to reduce the losses caused by disasters [13,14,15]. Therefore, increasing attention should be directed toward the mechanisms of these behaviors. However, although there are some studies on disaster risk management in rural areas in developed or developing countries (e.g., [13,16,17,18]), relatively few studies have paid attention to rural China, especially those rural areas where disasters and poverty exist together [19,20,21,22]. Compared with urban residents, rural residents, especially those in poor areas, are more vulnerable to the impact of disasters, especially those caused by earthquakes [8,11,19]. Therefore, it is more necessary to study the disaster risk management of this group, which can better guide them to deal with disasters and, thus, reduce the loss of life and property.

Disaster research has recently focused more on sociocultural factors. These disaster risk prevention and management factors include residents’ disaster risk perception, knowledge, and skills; disaster prevention and reduction awareness; disaster insurance purchase; and disaster prevention and relocation [11,15,18,23]. Studies of residents’ willingness to purchase disaster insurance and willingness to relocate to avoid disasters have generally considered factors such as residents’ knowledge and reported skills of disasters, disaster risk perception, and awareness of disaster risk reduction (e.g., [14,21,24,25]). For instance, based on the survey data of rural households in landslide-threatened areas of the Three Gorges Reservoir, Xu et al. [21,25] found that rural households’ disaster risk perception was significantly correlated with (had a direct influence on) their willingness to relocate and their disaster preparation. However, few studies have focused on the indirect effects of residents’ knowledge and reported skills of disasters, disaster risk perception, and awareness of disaster risk reduction on their willingness/behavior decision-making. Meanwhile, most studies have used descriptive statistical analysis combined with multiple linear regression, Logit, Probit, or Tobit tools to explore the correlations among residents’ knowledge and reported skills of disasters, perception of disaster risks, awareness of disaster risk reduction, and behavioral decision-making (willingness) (e.g., [21,22,25,26,27,28]). However, few studies have used the path analysis to systematically explore the interaction mechanisms among the above factors. Therefore, the process of residents’ behavior decision-making requires further examination.

Large-scale earthquake disasters have recently occurred in Sichuan [22]. For example, the Wenchuan earthquake in 2008 resulted in 124,600 casualties and direct economic losses of 0.85 trillion Yuan [6]. There were also numerous mudslide and landslide disasters surrounding the earthquake belts, which caused widespread poverty of rural households in mountain settlements [22]. The present study assessed the rural households located in areas severely affected by the 2008 Wenchuan earthquake. A systematic analysis was performed to create a model for the disaster risk perception of rural households. The following factors were considered using a path analysis method: the characteristics of rural households’ perception of disaster risks, awareness of disaster risk reduction, knowledge and reported skills of earthquake disasters, and willingness to avoid disasters. The study aimed to provide a reference for the formulation of relevant policies in the development of a resilient disaster prevention system in earthquake-threatened areas of Sichuan Province.

## 2. Theoretical Background 

Faced with the threat of disaster, residents will make behavioral decisions or have the willingness to make behavioral decisions. Empirical studies have shown that residents’ disaster risk perception is closely related to their behavioral decisions or behavioral decision-making willingness (e.g., [1,22,25,29,30]). For example, Xu et al. [22] found that the probability and severity of disasters were significantly correlated with the intention to purchase earthquake insurance, and Peng et al. [1] showed that the probability and fear of disasters were significantly correlated with the intention to purchase disaster insurance. In addition, Xu et al. [25] found a significant positive correlation between the perception of possibility, threat, and controllability of disasters and residents’ willingness to relocate. 

Meanwhile, in addition to disaster risk perception, residents’ awareness of disaster risk reduction is also an important factor influencing their behavioral decisions (e.g., [1]). Faced with the threat of disaster, residents in vulnerable areas generally lack the required ability to acquire disaster knowledge and skills [8]. The government, as an official channel of disaster information and one of the main providers of robust disaster prevention systems, is the source of residents’ dependence [31]. Therefore, for most residents, “attitude toward the government” also influences their behavioral decisions. For example, Peng et al. [1] found that trust in public institutions was significantly correlated with residents’ willingness to purchase disaster insurance. In addition, in the construction of disaster risk management and resilient disaster prevention systems, residents with a stronger sense of “subject responsibility consciousness” were generally active in risk management and had better disaster prevention preparation [32]. In addition, residents’ “disaster prevention tendency” will also affect their behavioral decisions. Faced with the threat of disaster, residents participate in training organized by the community to learn disaster risks, prevention, and reduction strategies to allow them to make better behavioral decisions [8,30,33,34].

Additionally, many empirical studies have shown that residents’ behavioral decisions, such as their willingness to purchase insurance or relocate, are closely related to their disaster knowledge and skills (e.g., [34]). Generally, residents who have greater knowledge and skills in disasters will exhibit greater willingness to purchase disaster insurance and relocate, because these actions can reduce the chances of being affected by a disaster [32].

**Figure 1:**
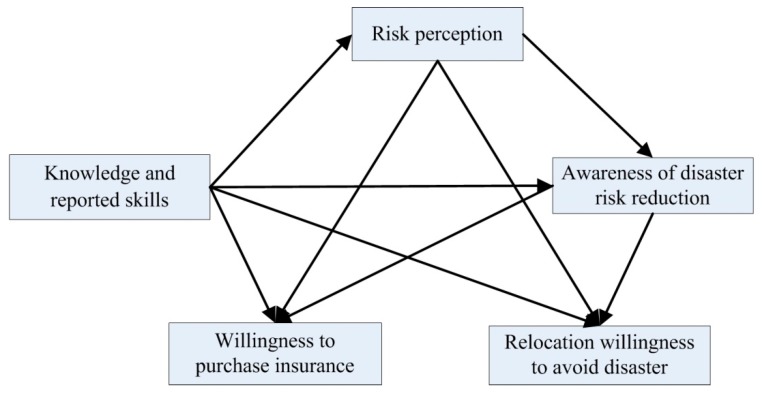
Theoretical framework of the disaster risk perception model.

Based on this, the present study proposed hypothesis H1:

**Hypothesis 1** **(H1).** *Residents’ disaster risk perception, residents’ awareness of disaster risk reduction, and residents’ knowledge and reporting skills are positively correlated with their willingness to purchase disaster insurance and their willingness to relocate (Figure 1)*.

With an increase of residents’ knowledge and skills in the face of disaster threats, their perceptions of disaster risk and awareness of disaster prevention/reduction will change, along with their behavioral decisions. For example, some residents acquired disaster knowledge from the internet and other sources and learned evacuation skills through training organized by villages. In this process, residents’ awareness of disaster prevention/reduction can be further strengthened, and they may be more inclined to purchase disaster insurance or relocate. Based on these factors, the research hypotheses H2 was proposed:

**Hypothesis 2** **(H2).** *The knowledge and skills acquired by residents will indirectly affect their willingness to purchase disaster insurance or relocate through disaster risk perception and awareness of disaster risk reduction, and residents’ disaster risk perception will indirectly affect their willingness to purchase disaster insurance or relocate through their awareness of disaster risk reduction (Figure 1)*.

## 3. Data and Methods

### 3.1. Data Source

The present study primarily used data from a questionnaire survey conducted in September 2015 in locations severely affected by the 2008 Wenchuan earthquake. Due to the fact that the survey assessed information such as the rural household’s annual income, factual issues regarding rural household’s information were investigated up to the end of 2014, while the current status of individual disaster risk perception and other psychological aspects were examined. Using stratified sampling, sample counties (cities) were selected from the 10 areas most severely affected by the Wenchuan earthquake.

**Figure 2:**
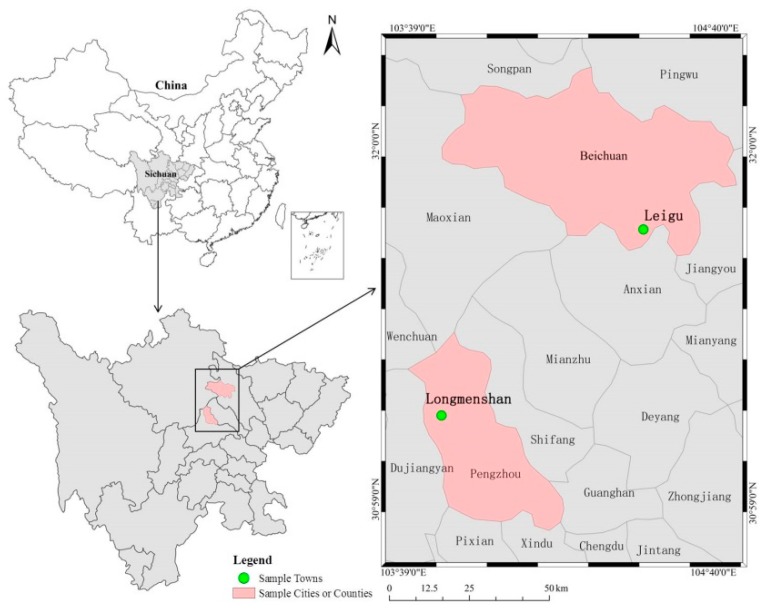
Locations of sample counties (cities) and sample towns.

Specifically, according to the differences in economic development levels, the 10 most severe disaster counties (cities), namely Wenchuan County and Mao County in Aba Prefecture, Beichuan County, An County and Pingwu County in Mianyang, Mianzhu City and Shifang City in Deyang, Qingchuan County in Guangyuan, and Dujiangyan City and Pengzhou City in Chengdu, were randomly divided into two groups, and one county (city) was randomly selected from each group to represent the sample county (city). Beichuan County (representing a low economic development level, with a per capita GDP of 17,052 Yuan in 2013, where 162,047 people were affected by the Wenchuan earthquake) and Pengzhou City (representing a high economic development level, with a per capita GDP of 30,736 Yuan in 2013, where 400,000 people were affected by the Wenchuan earthquake) were selected as the sample counties. Next, taking full account of the disaster severity of the sample counties (cities), together with the differences in economic and social development levels and ethnic distribution, this study selected Leigu Town in Beichuan Qiang Autonomous County and Longmenshan Town in Pengzhou City as the sample towns (according to statistics, the two sample towns were seriously affected by the Wenchuan earthquake in 2008, and the collapse rates of houses in these two towns were both over 75%) (Figure 2). Among them, Leigu Town (including 30 villages and 18,229 people who were affected by the Wenchuan earthquake) is located in the southeast of Beichuan County, and the Han people and Qiang ethnic groups live together in this territory. The terrain gradually decreases from west to east, and there is a complex geological environment and frequent mountain disasters. Leigu Town is about 60 km away from downtown Mianyang. Longmenshan Town (including 6 villages and 11,494 people who were affected by the Wenchuan earthquake) is located in the northern mountainous area of Pengzhou and in the transition zone of the Longmenshan fault zone. This town has numerous mountains, great altitude differences, abundant tourism resources, a high economic development level, and is 38 km away from downtown Pengzhou. Finally, according to the differences in the economic development level of villages, 4–5 villages were randomly selected from the towns to act as sample villages, and 20–36 rural households were randomly selected from these towns to act as the sample rural households to participate in the final investigation. After the sample rural households were determined, a person who knows about the household situation was randomly selected from the household for investigation and interview. On average, there were about four people living in each household. In the course of the investigation, investigators with enumeration and sampling skills, and who were well versed in the study context, were involved in data collection. After data entry, the results were collated and summarized. A total of 248 questionnaires were obtained from nine sample villages in two sample towns, and after the invalid questionnaires were excluded, a total of 241 valid questionnaires (117 from Pengzhou and 124 from Beichuan) were obtained. The effective rate of the questionnaire was 97.18% and the response rates was 99.2% (two rural households refused to be surveyed).

In this sample of 241 rural households, the proportion of male and female respondents accounted for 42.7% and 57.3% of the total sample, respectively. Respondents were mainly middle-aged and elderly; middle-aged people aged between 40 and 65 years accounted for 61.8%, and elderly people aged over 65 years made up 25.7% of the total sample. This age distribution was similar to that found in China’s mountainous rural settlements. The overall level of education of the respondents was low, with 23.2% reported to be illiterate. The majority of respondents had a primary school level education (nearly half), followed by junior high school level (24.9%) and senior high school level or above (5.4%).

### 3.2. Selection and Definition of Model Variables

#### 3.2.1. Rural Households’ Risk Perception Measurement

Disaster risk perception refers to the process in which individuals make subjective judgments on the characteristics and severity of disaster risks [5,20]. Mainly using the examples of quantitative methods in psychological paradigms, this study proposed that disaster risk perception is subjective and measurable. Referring to the research of Slovic [35], Lawrence et al. [36], Lindell and Hwang [37], Lindell [38], Lindell and Perry [39], Lindell and Whitney [40], and Xu et al. [21,22,25] on the measurement of disaster risk perception, this study intended to measure rural households’ level of disaster risk perception from four dimensions: “possibility”, “worry”, “controllability”, and “resilience”. Possibility refers to people’s assessment of the possibility of earthquake recurrence; worry describes people’s psychological and emotional feelings about earthquake disasters, which are characterized by fear and other psychological feelings; controllability indicates people’s uncontrollable perception of the negative effects of earthquakes and other chain disasters; and resilience represents the degree of people’s perception of the negative influence of an earthquake following a previously large earthquake (Table 1).

#### 3.2.2. Measurement of Rural Households’ Awareness of Disaster Risk Reduction

Examination of rural households’ awareness of disaster risk reduction was similar to that of rural households’ perception of disaster risks. Referring to the research by Hernández-Moreno and Alcántara-Ayala [27], Hori and Shaw [41], and Xu et al. [21] on the measurement of rural households’ awareness of disaster risk reduction, the present study designed 10 entries and measured rural households’ awareness of disaster risk reduction from three dimensions: subject responsibility consciousness, attitude toward government, and disaster prevention tendency (Table 2). Subject responsibility consciousness means that people reflect a sense of ownership and responsibility in disaster prevention and mitigation (i.e., self-rescue and mutual rescue during a disaster and assisting in the recovery and reconstruction after a disaster). Attitude toward government indicates people’s evaluation of the government’s work in disaster prevention and mitigation, and their confidence and trust in the government. Disaster prevention tendency refers to people’s actual behavioral tendency in disaster prevention and mitigation in daily life.

#### 3.2.3. Measurement of Rural Households’ Knowledge and Reported Skills of Earthquake Disasters

As shown in Table 3, the present study measured rural households’ knowledge and reported skills of earthquake disasters from three aspects: “knowledge of earthquake occurrence”, “knowledge of house safety”, and “skills of self-rescue and mutual rescue”. Higher scores in these three aspects indicated that people had a better understanding of the knowledge and reported skills of earthquake disasters.

#### 3.2.4. Measurement of Rural Households’ Willingness to Purchase Insurance and Willingness to Avoid Disasters and Relocate

This study mainly measured rural households’ willingness to purchase insurance and willingness to avoid disasters and relocate through the following two questions. The possible answers to the question were: 1 = strongly unwilling, 2 = unwilling, 3 = neutral, 4 = willing, 5 = strong willing.

(1) If there is earthquake disaster insurance, would you be willing to purchase it?
(2) If you were given some protection (such as a large subsidy), would you be willing to relocate to avoid disasters?

## 4. Results

### 4.1. Descriptive Statistical Analysis of Model Variables

#### 4.1.1. Rural Households’ Risk Perception

The present study initially tested the reliability of 13 entries in Table 1 to measure rural households’ level of disaster risk perception. The results showed that the Cronbach α value of the internal consistency of all entries was 0.73, and the Cronbach α values of possibility, worry, controllability, and resilience were 0.79, 0.79, 0.82, and 0.62, respectively, indicating that the internal consistency of the entries was good (Table 4). Confirmatory factor analysis was used to perform dimensional reduction analysis on the 13 entries. The Kaiser–Meyer–Olkin (KMO) test value was 0.70 and the cumulative variance contribution rate of the factor analysis was 64.95%. In addition, comprehensive scores of possibility, worry, controllability, and resilience were obtained (Table 4). Finally, referring to the research from Xu et al. [21,22,25], the present study used the efficiency coefficient method to convert the scores of the above four dimensions into percentage scores. Further information on the efficacy coefficient method, formula, and calculations are provided in the appendix of Xu et al. [22]. 

#### 4.1.2. Rural Households’ Awareness of Disaster Risk Reduction

The results of the reliability test showed that the Cronbach α value of the internal consistency of 10 entries in Table 2 was 0.66, and the Cronbach α values of subject responsibility consciousness, attitude toward government, and disaster prevention tendency were 0.63, 0.60, and 0.67, respectively. This indicated that the internal consistency of the entries was good and the entries were suitable for subsequent analysis (Table 5). The confirmatory factor analysis was also used to conduct a dimensional reduction analysis on the 10 entries. The KMO test value was 0.68 and the cumulative variance contribution rate of the factor analysis was 55.14%. The comprehensive scores of subject responsibility consciousness, attitude toward government, and disaster prevention tendency were obtained (Table 5). Furthermore, the present study used the efficiency coefficient method to convert the scores of the above three dimensions into percentage scores.

#### 4.1.3. Rural Households’ Knowledge and Reported Skills of Earthquake Disasters

As shown in Table 3, 25% of the residents mastered knowledge of earthquake occurrence, 87% of the residents mastered knowledge of house safety, 58% of the residents mastered skills of self-rescue and mutual rescue, and 57% of the residents mastered comprehensive knowledge and skills.

#### 4.1.4. Rural Households’ Willingness to Purchase Insurance and Willingness to Avoid Disasters and Relocate

Table 6 presents the frequency distribution of rural households’ willingness to purchase insurance and willingness to relocate to avoid disasters. The results showed that 53.11% of rural households located in areas severely affected by the Wenchuan earthquake were very willing to purchase insurance, while only 15.35% of the respondents were reluctant. In addition, 72.19% of rural households were very willing to relocate, whereas only 5.39% were very reluctant.

### 4.2. Correlation Coefficient Matrix of Variables in the Models

Table 7 presents the correlation coefficient matrix of variables in the models. As shown in Table 7, the correlation coefficients between all variables were less than 0.5, indicating that there was no serious multicollinearity among the model independent variables. Respondents’ willingness to purchase disaster insurance was positively significantly correlated with subject responsibility consciousness, disaster prevention tendency, controllability, knowledge of earthquake occurrence, knowledge of house safety, and skills of self-rescue and mutual rescue, and was negatively significantly correlated with age. Respondent’s willingness to relocate was positively significantly correlated with disaster prevention tendency, possibility, and knowledge of earthquake occurrence, and was negatively significantly correlated with resilience. The three dimensions of awareness of disaster risk reduction were significantly correlated with different dimensions of disaster risk perception, knowledge and reported skills, and some socioeconomic indicators of respondents. For example, attitude towards government was positively significantly correlated with resilience, skills of self-rescue and mutual rescue, and age, and was negatively significantly correlated with experience. The four dimensions of disaster risk perception were significantly correlated with different dimensions of knowledge and reported skills and some socioeconomic indicators of respondents. For example, worry was positively significantly correlated with knowledge of house safety, and was negatively significantly correlated with gender and education. The three dimensions of knowledge and reported skills were significantly correlated with some socioeconomic indicators of respondents. For example, knowledge of earthquake occurrence was negatively significantly correlated with age and nationality, and was positively significantly correlated with education and experience.

### 4.3. Analysis of Basic Characteristics of Model Variables

**Figure 3:**
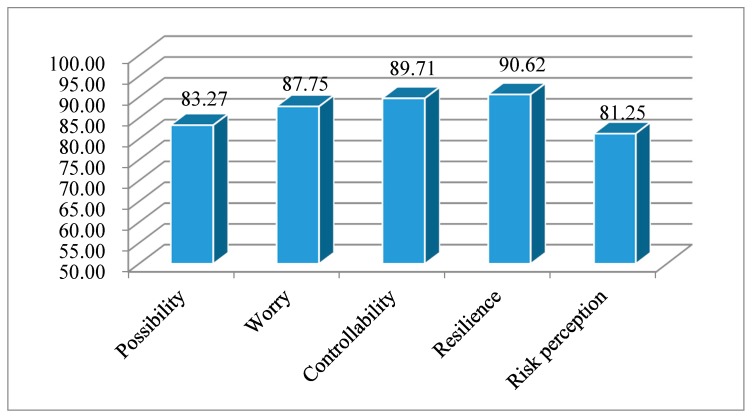
Scores of rural households’ risk perception.

Figure 3 shows the scores of rural households’ perception of disaster risks. Given their close proximity to areas affected by the Wenchuan earthquake, the rural households’ perception of disaster risk scores were relatively high and the average scores of the four dimensions of the perception of disaster risks were all above 83. Resilience had the highest score (90.62 points), while possibility had the lowest score (83.27 points). The scores of controllability and worry were 89.71 points and 87.75 points, respectively. Most respondents were found to only remember the damage caused by large earthquakes, which caused them to fear the occurrence of future earthquakes. Many people also believed that following the Wenchuan earthquake, other large earthquakes would not occur during their lifetime; therefore, their perception of risk possibility was relatively low. 

With the publicity of earthquake disaster knowledge and skills of disaster prevention and mitigation, rural households had a higher perception in managing earthquake and secondary disasters and a lower perception of the negative influence of future earthquakes on their families.

**Figure 4:**
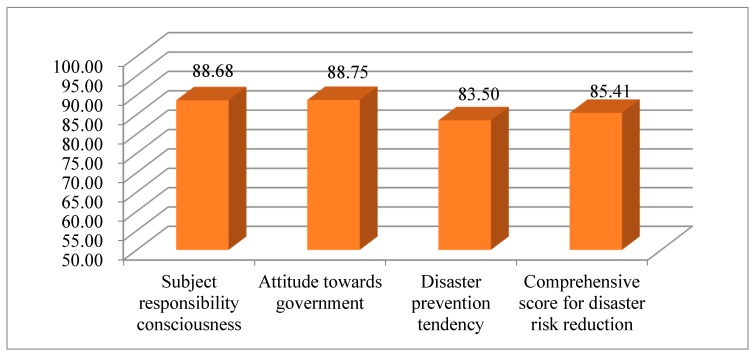
The scores of rural households’ awareness of disaster risk reduction.

Figure 4 presents the scores of rural households’ awareness of disaster risk reduction. Rural households had a stronger awareness of disaster risk reduction, and the average comprehensive scores of the three dimensions of rural households’ awareness of disaster risk reduction were above 83. Attitude toward government had the highest score (88.75 points). The score of residents’ support of the government’s actions during disasters was relatively high (the mean of B7 was 4.47). 

The score of subject responsibility consciousness was only 0.07 points lower than that of attitude toward government, and all entries averaged above 4.2. This suggested that after experiencing a large earthquake, people generally had a stronger subject responsibility consciousness and stronger responsibility tendency in disaster prevention, self-rescue, and mutual rescue during disasters, and recovery and reconstruction after disasters. Disaster prevention tendency had the lowest score (83.50 points), which included entries B9 and B10 that averaged below 3.6.

As shown in Table 5, the weighted average of the three dimension entries corresponding to each sample (the weights of entries within each dimension were equal), were used to obtain the comprehensive score of each dimension. The average score of knowledge of earthquake occurrence was 0.25; however, the C1 score showed that residents did not clearly understand how earthquakes occur. The average score of knowledge of house safety was 0.87, suggesting that people knew that house safety was a potentially significant safety hazard during earthquakes. The average score of skills of self-rescue and mutual rescue was 0.58. Overall, the average comprehensive score of knowledge and skills was 0.57. These results showed that seven years following the Wenchuan earthquake, through effective publicity and education, people had a certain degree of knowledge of earthquake occurrence and house safety, and skills in self-rescue and mutual rescue. 

### 4.4. Test of Disaster Risk Perception Process Model

**Figure 5:**
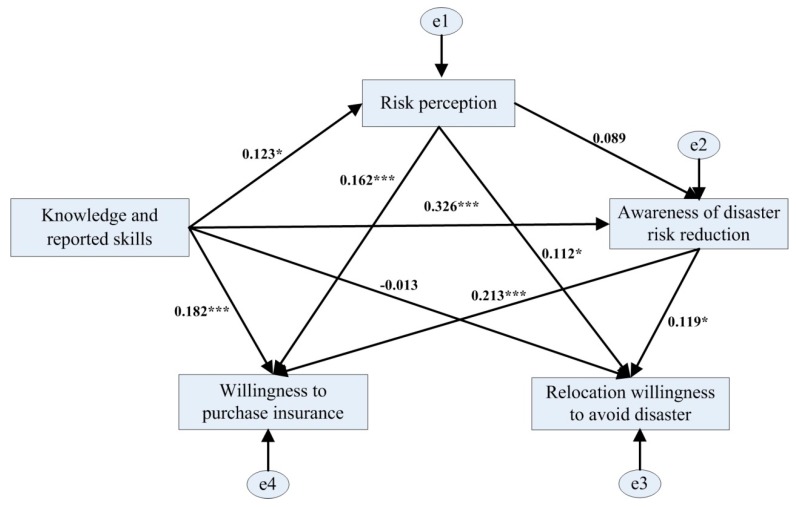
Path analysis results of the disaster risk perception model. Note: * and *** refer to *p* < 0.1 and *p* < 0.01, respectively.

Amos v17.0 was used to create the model for the process of disaster risk perception (Figure 5). The parameters next to the arrows in the model represent the magnitude of partial regression coefficients. The overall fitting results of the model were good (χ^2^ = 0.286, df = 1, *p* = 0.593, GFI = 1.000, AGFI = 0.993, RMSEA = 0.000). The coefficient test results were systematically analyzed as follows.

As shown in Figure 5 and Table 8, knowledge and skills was significantly positively correlated with willingness to purchase insurance, while the correlation between knowledge and skills and willingness to relocate to avoid disaster was not significant. The total effect between knowledge and skills and willingness to purchase insurance was 0.274, in which the direct effect was 0.182 and the indirect effect was 0.092. The indirect effect was mainly realized by the moderating effect of risk perception and awareness of disaster risk reduction (knowledge and skills → risk perception → willingness to purchase insurance; knowledge and skills → risk perception → awareness of disaster risk reduction → willingness to purchase insurance; knowledge and skills → awareness of disaster risk reduction → willingness to purchase insurance).

There were significant positive correlations between risk perception and willingness to purchase insurance, as well as willingness to relocate to avoid disasters. The total effect between risk perception and willingness to purchase insurance was 0.181, in which the direct effect was 0.162 and the indirect effect was 0.019. The indirect effect was mainly realized by the moderating effect of awareness of disaster risk reduction (risk perception → awareness of disaster risk reduction → willingness to purchase insurance). The total effect between risk perception and willingness to relocate to avoid disaster was 0.123, in which the direct effect was 0.112 and the indirect effect was 0.011. The indirect effect was mainly realized by the moderating effect of awareness of disaster risk reduction (risk perception → awareness of disaster risk reduction → willingness to relocate to avoid disaster).

There were significant positive correlations between awareness of disaster risk reduction and willingness to purchase insurance, as well as willingness to relocate to avoid disasters. The total effect between awareness of disaster risk reduction and willingness to purchase insurance was 0.213, in which the direct effect was 0.213 and the indirect effect was 0.000. The total effect between awareness of disaster risk reduction and willingness to relocate to avoid disaster was 0.119, in which the direct effect was 0.119 and the indirect effect was 0.000. 

## 5. Discussion

Based on the survey data of rural households located in areas severely affected by the Wenchuan earthquake, a model was created to analyze the characteristics of rural households’ knowledge and reported skills in earthquake disasters, level of disaster risk perception, awareness of disaster risk reduction, and willingness to avoid disasters. There are some similarities and differences between the results of this study and the findings of previous research. 

Except that knowledge and reported skills was not significantly correlated with relocation willingness to avoid disaster, consistent with hypothesis H1, this study found that residents’ disaster risk perception and awareness of disaster risk reduction were both significantly positively correlated with willingness to buy insurance and relocation willingness to avoid disaster, while knowledge and reported skills was significantly positively correlated with willingness to buy insurance. However, there are similarities and differences in the correlation between residents’ disaster risk perception, awareness of disaster risk reduction, and knowledge and reported skills, as well as willingness to buy insurance and relocation willingness to avoid disaster (e.g., Baker [29], Botzen et al. [42], Siegrist and Gutscher [43], Paul and Bhuiyan [44], Xu et al. [21,22,25], Turner et al. [45], Farley et al. [46], Showalter [47], Miceli et al. [48], Takao et al. [49], Thieken et al. [50]). For example, consistent with studies of Xu et al. [25], this study also found that in face of disaster threat, residents’ willingness to relocate was significantly positively correlated with the possibility perception of disaster occurrence; However, inconsistent with studies of Xu et al. [25], which found that the controllability perception of disasters was significantly negatively correlated with the relocation willingness, this study found that the controllability perception of disasters was not significantly correlated with the relocation willingness, which may be due to the different types of disasters. Furthermore, consistent with Dooley et al. [51], Farley et al. [46], Lindell and Perry [52], Showalter [47], and Xu et al. [22], this study also found that there was a significant positive correlation between disaster probability perception and disaster insurance purchase willingness. However, it was inconsistent with Lindell and Perry [52] and Xu et al. [21], who found no significant correlation between probability perception of disasters and insurance purchase willingness. In addition, consistent with hypothesis H2, this study found that rural households’ knowledge and reported skills of earthquake disasters, perception of disaster risks, and awareness of disaster risk reduction could directly and indirectly affect their willingness to purchase disaster insurance and willingness to relocate to avoid disasters.

In addition to the academic marginal contributions, the results of the present study have important policy implications for the creation of resilient disaster prevention systems in China. For instance, it was found that rural households’ located in areas severely affected by the Wenchuan earthquake had a stronger willingness to purchase earthquake disaster insurance and a stronger willingness to relocate to avoid disasters. Therefore, the government could consider strategies to introduce earthquake disaster insurance in such areas by assessing rural households’ requirements and willingness to afford the insurance costs. Through the implementation of earthquake disaster insurance, rural households could obtain a greater sense of security, so as to minimize problems caused by wanting to relocate to avoid disasters. In addition, it was observed that rural households’ knowledge and reported skills of earthquake disasters, perception of disaster risks, and awareness of disaster risk reduction could directly or indirectly affect their willingness to purchase disaster insurance and willingness to relocate to avoid disasters. Thus, the government could implement measures to improve rural households’ knowledge of earthquake disasters to inform better decision-making to reduce property losses.

The present study had several limitations, which could be addressed in future research. For example, it is unknown whether the research results and conclusions can be applied to other locations less threatened or affected by earthquakes, or to other types of disasters, such as mudslides and landslides. Rural households’ disaster risk perception is a dynamic process and panel data are required to reveal dynamically changing processes. However, the present study was only based on static cross-sectional data. Panel data could be used to further verify the conclusions of this study in future research. At the same time, this study focused on rural households in rural areas of developing countries. Whether the research conclusion is applicable to urban residents or residents in developed countries needs further verification. Even for China, a developing country, there may be many differences between urban residents and rural residents in education level, socioeconomic development level, disaster knowledge, disaster prevention and reduction skills, and awareness of disaster prevention and reduction, due to the differences in location and resource endowment. Future studies can further use the survey data of urban residents to verify whether the conclusion of this study is still valid. If not, we should find out the reasons for the differences and formulate corresponding disaster prevention and reduction policies according to the characteristics of different regions in urban and rural areas. Moreover, compared with the large-scale rural residents in the earthquake disaster areas, although this study obtained 241 effective questionnaires through stratified random sampling, the overall number was still relatively small. Future studies can further expand the sample size to test the results of this study. At the same time, the education level of the interviewees was generally low, which may affect their acquisition of disaster knowledge and disaster skills, and thus affect their behavioral decision-making or behavioral decision-making willingness. However, this is the reality of rural areas in rural China. Driven by urbanization, a large number of young labor forces go out for work and the elderly live in rural areas for a long time. In the future, the research object can be further expanded, and information about disaster risk management of migrant workers can be obtained through telephone surveys, so as to better provide reference for the formulation of disaster prevention and reduction policies. After all, faced with the threat of disaster, the actual decision-making of the family’s disaster avoidance behavior is more the result of the collective discussion of all family members. In addition, this study only focused on the relationships between residents’ risk perceptions, knowledge, and willingness related to disasters, rather than residents’ actual behavioral decision-making. Although some studies (e.g., [53,54,55,56]) have shown a significant positive correlation between residents’ willingness and their actual behavioral decision-making, future studies are required to verify whether this is true for the present study.

## 6. Conclusions

The following conclusions were obtained from the present study:

(1) Rural households’ perception of disaster risks consisted of four dimensions: possibility, worry, controllability, and resilience. Rural households’ awareness of disaster risk reduction was composed of three dimensions: subject responsibility consciousness, attitude toward government, and disaster prevention tendency. Rural households’ knowledge and reported skills of earthquake disasters included three dimensions: knowledge of earthquake occurrence, knowledge of house safety, and skills of self-rescue and mutual rescue.
(2) Of the people surveyed, 53.11% had a stronger willingness to purchase disaster insurance and 72.19% had a stronger willingness to relocate to avoid disasters.
(3) Risk perception, knowledge and skills, and awareness of disaster risk reduction were significantly correlated with willingness to purchase insurance, and the total effects were 0.181, 0.274, and 0.213, respectively. Risk perception and awareness of disaster risk reduction were significantly positively correlated with willingness relocate to avoid disaster, and the total effects were 0.123 and 0.119, respectively. Knowledge and skills could indirectly affect willingness to purchase insurance and willingness to relocate to avoid disaster through risk perception and awareness of disaster risk reduction. Risk perception could indirectly affect willingness to purchase insurance and willingness to relocate to avoid disaster through awareness of disaster risk reduction.

## Figures and Tables

**Table 1:** Risk perception measurement.

Code	Item ^a^	Mean	SD ^b^
A1	In the next 10 years, there will be earthquakes near my home.	3.05	1.11
A2	I think the risk of earthquake disaster is increasing here in recent years.	3.19	1.19
A3	We have a greater risk of earthquakes here than in other regions.	3.51	1.17
A4	I think our family’s health will be affected by earthquakes in the next 10 years.	3.18	1.19
A5	I believe my housing and land will be greatly affected by earthquakes in the next 10 years.	3.14	1.14
A6	I always feel that an earthquake will come eventually.	3.12	1.32
A7	When I think of an earthquake, I feel afraid.	3.20	0.83
A8	I’m worried about the impact of the earthquake on the village and my family.	4.42	1.12
A9	In the event of a disaster, I feel like the world is ending.	4.40	1.03
A10	Although the earthquake is not controllable, I can perform some measures (such as strengthening the house) to reduce any losses.	3.98	1.21
A11	There are some approaches, such as governance, that can reduce other disasters caused by earthquakes.	4.27	0.94
A12	I feel that my house is stronger than it was in 2008, and in the event of disasters, the losses will be smaller.	1.80	0.90
A13	I feel that my family will recover faster and better in the event of an earthquake compared to 2008.	1.80	0.75

^a^ 1 = totally disagree, 2 = disagree, 3 = neutral, 4 = agree, 5 = totally agree; ^b^ SD = standard deviation.

**Table 2:** Measurement of awareness of disaster risk reduction.

Code	Item ^a^	Mean	SD ^b^
B1	I usually tell my children to pay attention to their safety and prevent disasters.	4.59	0.67
B2	When some of my relatives and friends are affected by disasters, I am willing to help them.	4.66	0.61
B3	I think I should be responsible for the safety of my family during disasters.	4.54	0.63
B4	After disasters, I actively perform recovery and reconstruction activities.	4.24	0.85
B5	I think the disaster drills organized by the government are very effective.	3.97	1.07
B6	I think the judgment of the government on disasters is credible.	4.32	1.00
B7	I support the government’s actions during disasters.	4.47	0.68
B8	Due to the performance of the government and related agencies during earthquakes, I have more confidence in my family’s ability to cope with disasters.	4.248	0.84
B9	I usually have the awareness to learn some skills of disaster prevention and mitigation.	3.11	1.30
B10	I usually actively participate in disaster management publicity and training events.	3.54	1.24

^a^ 1 = totally disagree, 2 = disagree, 3 = neutral, 4 = agree, 5 = totally agree; ^b^ SD = standard deviation.

**Table 3:** The entries related to knowledge and reported skills of earthquake disasters.

Code	Item	Mean	SD ^a^
C1	Do you know exactly how earthquake disasters occur? (0 = No, 1 = Yes)	0.13	0.34
C2	Do you think earthquakes may be caused by Land Bodhisattvas or God? (0 = No, 1 = Yes)	0.22	0.41
C3	Do you think earthquakes are caused by movement in the Earth’s crust? (0 = No, 1 = Yes)	0.53	0.50
Knowledge of earthquake occurrence: (C1 + C2 + C3)/3		0.25	0.19
C4	Do you pay attention to the surrounding geological conditions when choosing land/housing? (0 = No, 1 = Yes)	0.94	0.24
C5	Do you think about the stability of the foundation of your house? (0 = No, 1 = Yes)	0.93	0.42
C6	Will you consider reinforcing your house to prevent it from being damaged during earthquakes? (0 = No, 1 = Yes)	0.73	0.44
Knowledge of house safety: (C4 + C5 + C6)/3		0.87	0.26
C7	During an earthquake, do you know how to escape? (0 = No, 1 = Yes)	0.61	0.49
C8	After an earthquake disaster, will you consider whether it is safe to immediately return to your house? (0 = No, 1 = Yes)	0.57	0.50
C9	When an earthquake occurs and people are trapped, do you know how to rescue them? (0 = No, 1 = Yes)	0.56	0.50
Skills of self-rescue and mutual rescue: (C7 + C8 + C9)/3		0.58	0.32
Comprehensive knowledge and skills: (C1 + C2 + C3 + C4 + C5 + C6 + C7 + C8 + C9)/9		0.57	0.16

^a^ SD = standard deviation.

**Table 4:** Component matrixes of risk perception after rotation.

Items	Factors			
	Possibility	Worry	Controllability	Resilience
A1	**0.75**	−0.01	−0.01	0.06
A2	**0.75**	−0.14	−0.06	0.14
A3	**0.65**	0.14	−0.11	−0.11
A4	**0.70**	0.18	0.06	0.01
A5	**0.69**	0.19	0.06	0.05
A6	**0.56**	0.20	0.07	0.22
A7	0.06	**0.92**	−0.00	0.02
A8	0.08	**0.89**	−0.06	0.01
A9	0.25	**0.65**	−0.14	−0.06
A10	−0.04	−0.15	**0.90**	0.08
A11	0.05	−0.03	**0.93**	0.06
A12	0.05	0.00	0.04	**0.84**
A13	0.12	−0.03	0.08	**0.83**
Eigenvalue	2.96	2.25	1.73	1.51
Explained variance	22.75	17.29	13.32	11.58
Cumulative variance	22.75	40.04	53.36	64.95
Cronbach α	0.79	0.79	0.82	0.62

In each component, the bold numbers represent the component is mainly composed by the corresponding items.

**Table 5:** Component matrixes of awareness of disaster risk reduction after rotation.

Items	Factors		
	Subject Responsibility Consciousness	Attitude Towards Government	Disaster Prevention Tendency
B1	**0.64**	0.05	0.02
B2	**0.70**	0.05	0.00
B3	**0.71**	0.09	−0.08
B4	**0.70**	0.01	0.17
B5	0.05	**0.45**	0.46
B6	−0.21	**0.59**	0.23
B7	0.21	**0.76**	0.08
B8	0.16	**0.80**	0.01
B9	0.02	−0.03	**0.85**
B10	0.05	0.27	**0.80**
Eigenvalue	2.00	1.84	1.67
Explained variance	19.99	18.41	16.74
Cumulative variance	19.99	38.40	55.14
Cronbach α	0.63	0.60	0.67

In each component, the bold numbers represent the component is mainly composed by the corresponding items.

**Table 6:** The frequency distribution table of rural households’ willingness to purchase insurance and willingness to avoid disasters and relocate.

Code	Item ^a^	Option	Frequency (%)
D1	If there is earthquake disaster insurance, would you be willing to purchase it?	1	7.05
		2	8.30
		3	31.54
		4	18.67
		5	34.44
D2	If you were given some protection (such as a large subsidy), would you be willing to relocate to avoid disasters?	1	5.39
		2	16.18
		3	6.22
		4	35.68
		5	36.51

^a^ 1 = strongly unwilling, 2 = unwilling, 3 = neutral, 4 = willing, 5 = strong willing.

**Table 7:** Correlation coefficient matrix of variables in the models.

Variable	1	2	3	4	5	6	7	8	9	10	11	12	13	14	15	16	17	18
1	1																	
2	0.087	1																
3	0.224 ***	0.092	1															
4	0.089	0.005	0.000	1														
5	0.198 ***	0.131 **	0.000	0.000	1													
6	0.190 ***	0.240 ***	0.020	0.043	−0.121 *	1												
7	0.035	−0.021	0.064	−0.066	−0.040	0.000	1											
8	0.127 **	0.029	0.324 ***	−0.014	−0.062	0.000	0.000	1										
9	0.044	−0.108 *	0.219 ***	0.210 ***	0.024	0.000	0.000	0.000	1									
10	0.140 **	0.110 *	−0.033	−0.024	0.113 *	0.082	−0.031	−0.127 **	−0.198 ***	1								
11	0.142 **	−0.007	0.132 **	−0.093	0.150 **	−0.060	0.129 **	0.334 ***	0.085	−0.078	1							
12	0.197 ***	0.000	0.281 ***	0.131 **	0.253 ***	0.019	−0.048	0.059	0.176 ***	0.101	0.019	1						
13	−0.076	−0.069	0.172 ***	0.022	0.105	−0.127 **	−0.256 ***	0.060	0.082	−0.101	0.015	0.208 ***	1					
14	−0.116 *	0.013	−0.101	0.228 ***	−0.323 ***	−0.045	−0.051	−0.028	0.086	−0.232 ***	−0.172 ***	−0.021	0.018	1				
15	0.080	0.027	0.169 ***	−0.062	0.373 ***	0.002	−0.156 **	0.024	0.085	0.142 **	0.116 *	0.191 ***	0.277 ***	−0.560 ***	1			
16	−0.056	0.030	0.166 ***	0.065	0.081	−0.172 ***	−0.044	0.031	0.246 ***	−0.175 ***	0.070	0.001	0.119 *	0.132 **	0.068	1		
17	−0.060	0.078	0.085	−0.113 *	0.147 **	0.059	0.026	−0.083	−0.037	0.107 *	−0.018	0.004	0.114 *	−0.200 ***	0.199 ***	−0.002	1	
18	0.093	−0.01	0.188 ***	0.015	−0.021	0.015	−0.001	0.027	−0.011	0.122 *	−0.116 *	0.124 *	0.098	−0.145 **	0.210 ***	−0.134 **	0.077	1

Note: *** *p* < 0.01, ** *p* < 0.05, * *p* < 0.1; 1 = willingness to purchase insurance; 2 = willingness to relocate; 3 = subject responsibility consciousness; 4 = attitude towards government; 5 = disaster prevention tendency; 6 = possibility; 7 = worry; 8 = controllability; 9 = resilience; 10 = knowledge of earthquake occurrence; 11 = knowledge of house safety; 12 = skills of self-rescue and mutual rescue; 13 = gender; 14 = age; 15 = education; 16 = nationality; 17 = experience; 18 = income.

**Table 8:** The results of total effects, direct effects, and indirect effects.

Effect Pathway	Total Effects	Direct Effects	Indirect Effects
Risk perception <--- Knowledge and reported skills	0.123 *	0.123	0.000
Awareness of disaster risk reduction <--- Knowledge and reported skills	0.337 ***	0.326	0.011
Willingness to buy insurance <--- Knowledge and reported skills	0.274 ***	0.182	0.092
Relocation willingness to avoid disaster <--- Knowledge and reported skills	0.041	−0.013	0.054
Awareness of disaster risk reduction <--- Risk perception	0.089	0.089	0.000
Willingness to buy insurance <--- Risk perception	0.181 ***	0.162	0.019
Relocation willingness to avoid disaster<--- Risk perception	0.123 *	0.112	0.011
Willingness to buy insurance <--- Awareness of disaster risk reduction	0.213 ***	0.213	0.000
Relocation willingness to avoid disaster <---Awareness of disaster risk reduction	0.119 *	0.119	0.000

Note: The data in the table are all standardized data; * and *** refer to *p* < 0.1 and *p* < 0.01, respectively.
